# Methylpalladium complexes with pyrimidine-functionalized N-heterocyclic carbene ligands

**DOI:** 10.3762/bjoc.12.150

**Published:** 2016-07-21

**Authors:** Dirk Meyer, Thomas Strassner

**Affiliations:** 1 Physikalische Organische Chemie, TU Dresden, Bergstraße 66, 01062 Dresden, Germany

**Keywords:** alkane activation, alkyl complex, NHC, palladium, solid state structure

## Abstract

A series of methylpalladium(II) complexes with pyrimidine-NHC ligands carrying different aryl- and alkyl substituents R ([((pym)^(NHC-R))Pd^II^(CH_3_)X] with X = Cl, CF_3_COO, CH_3_) has been prepared by transmetalation reactions from the corresponding silver complexes and chloro(methyl)(cyclooctadiene)palladium(II). The dimethyl(1-(2-pyrimidyl)-3-(2,6-diisopropylphenyl)imidazolin-2-ylidene)palladium(II) complex was synthesized via the free carbene route. All complexes were fully characterized by standard methods and in three cases also by a solid state structure.

## Introduction

Palladium complexes have been shown to be versatile homogeneous catalysts in a variety of reactions [1]. One of the most prominent examples are the palladium catalyzed cross-coupling reactions [2], but in addition palladium complexes recently received much attention also in the field of selective CH oxidations [3–6]. The catalytic conversion of alkanes and especially of methane into a value-added products while avoiding over-oxidation to carbon dioxide is still one of the most difficult tasks and considered to be one of the “Holy Grails” of chemistry [7]. Early work by Bergman [8–9] and Graham [10–11] showed that the activation of methane is possible, but their systems provided only stoichiometric reactions. The Shilov system [12–17], had the disadvantage of using stoichiometric amounts of platinum for the reoxidation of the active catalyst and the platinum bispyrimidine system [18–20] was not successful because of the large amounts of diluted sulfuric acids as the byproduct of the methanol synthesis. Sen reported the activity of palladium(II) catalysts for the oxidation of methane [21] and several groups contributed to the progress in the field which is summarized in recent reviews [22–23]. The system based on N-heterocyclic carbene (NHC) ligands developed in our group uses a mixture of trifluoroacetic acid (TFA) and its anhydride (TFAA) as solvents. It has the advantage that the formed ester can be separated and hydrolyzed, followed by recycling of the free acid which potentially can be reused in the process [24–30]. Contrary to the “Catalytica” bispyrimidine system, for the biscarbene system the palladium complexes turned out to be more stable than the corresponding platinum complexes under the reaction conditions [31–32], although Peter Hofmann had recently shown the stability and reactivity of the platinum-alkyl complexes with bis-NHC ligand [33]. To study the differences between both systems we synthesized palladium [34] and platinum [35] “hybrid complexes” with ligands combining both structural elements the pyrimidine as well as the NHC fragment. We also used density functional theory (DFT) calculations to investigate the mechanism and potential intermediates.

Quantum chemical (QC) investigations have been very helpful during the last years in elucidating the mechanisms of palladium-catalyzed reactions [26,36]. According to our QC calculations the catalytic cycle for the alkane activation by bis(NHC) palladium complexes like the bis(1,1'-dimethyl-3,3'-methylenediimidazoline-2,2'-diylidene)palladium(II) dibromide [L_2_PdBr_2_] consists of three steps: electrophilic substitution, oxidation and reductive elimination involving a palladium(IV) intermediate [26]. But we also experimentally set out to investigate potential intermediates of the catalytic cycle [37]. One of the proposed intermediates in the catalytic cycle is a monomethyl complex which is formed from the starting material after methane activation and which could either carry a bromo [L_2_PdBr(CH_3_)] or a trifluoroacetato [L_2_Pd(CF_3_COO)(CH_3_)] ligand. These intermediates should be active according to the proposed catalytic cycle, which starts with an electrophilic substitution reaction. We therefore set out to synthesize the corresponding methyl complexes for the “hybrid ligand” with chloro- and trifluoroacetato ligands.

## Results and Discussion

### Synthesis

**Scheme 1:**
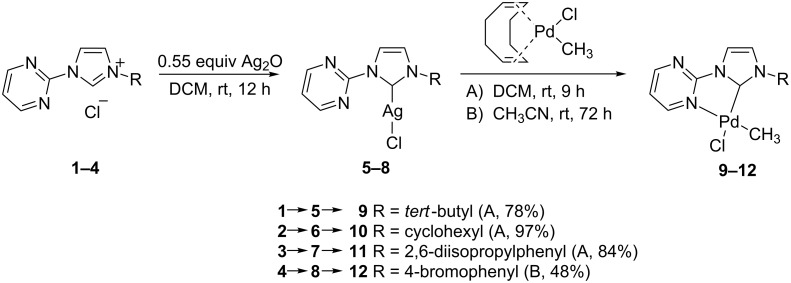
Synthesis of the monomethylpalladium(II) complexes **9**–**11** (in DCM) and **12** (in CH_3_CN).

Some time ago we reported the synthesis of the imidazolium salts **1**–**4** and their corresponding silver complexes **5**–**8**. The reaction with dichloro(cyclooctadiene)-palladium(II) [(COD)Pd^II^Cl_2_] allowed for the isolation and characterization of the [((pym)^(NHC-R))Pd^II^Cl_2_]-complexes [34]. For the synthesis of the corresponding monomethyl complexes we could rely on earlier work by Byers and Canty which reported the synthesis of methyl- and dimethylpalladium(II) complexes with various nitrogen donor systems [38]. The palladium complexes [(pym)^(NHC-R)Pd^II^(CH_3_)Cl] **9**–**12** have been synthesized in good yields from the corresponding silver complexes **5**–**8** by transmetalation with chloro(methyl)(cyclooctadiene)palladium(II) [(COD)Pd^II^(CH_3_)Cl] either in dichloromethane (A) or acetonitrile (B, Scheme 1).

**Figure 1:**
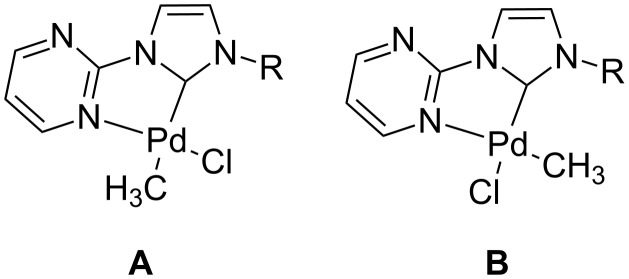
Possible isomers.

Although two isomers could be formed we only observed the isomer **B**, where the methyl group is located *cis* to the carbene carbon atom (Figure 1). This can be explained by the strong donor character of the carbene which makes the *trans* isomer less favorable. NMR analysis of the reaction products confirms that only one complex is formed. Additional proof comes from density functional theory (DFT) calculations which predict isomer **B** to be more stable for all complexes **9**–**12**. At the B3LYP/6-311+G** level of theory the isomer with the methyl group coordinated *cis* to the carbene carbon atom the **B** isomers are thermodynamically favored by 5–11 kcal/mol (Table 1).

**Table 1:** Calculated free energy differences (Δ*G* in kcal/mol) between the *trans-* (**A**) and *cis-* (**B**) isomers (B3LYP/6-311+*G*(d,p)).

Complex	Substituent R	Δ*G*

**9**	*tert-*butyl	4.7
**10**	cyclohexyl	7.3
**11**	diisopropylphenyl	10.7
**12**	4-bromophenyl	10.0

**Figure 2:**
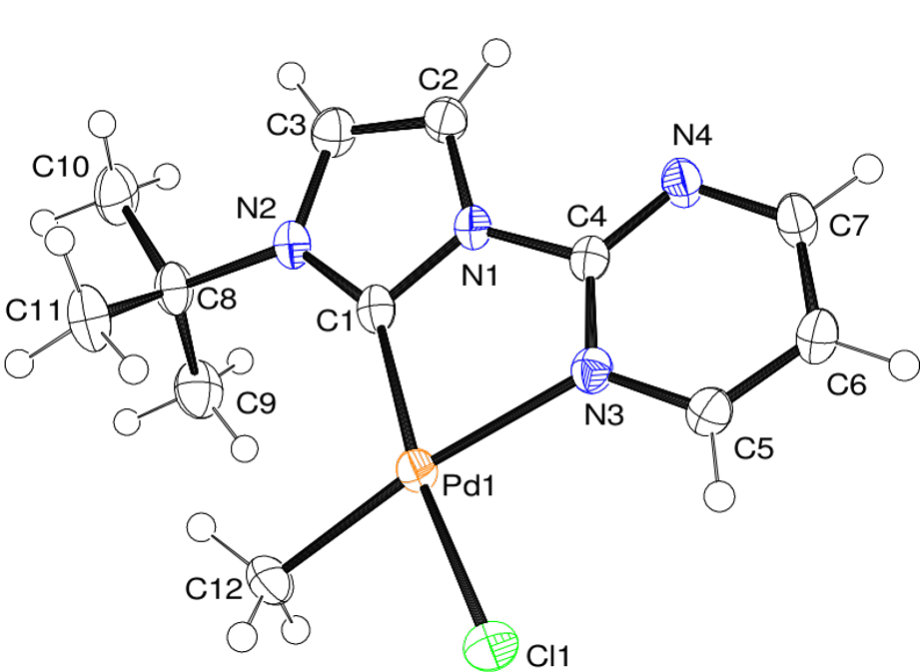
ORTEP [39] style plot of complex **9** in the solid state. Thermal ellipsoids are given at the 50% probability level. Selected bond lengths in angstrom and angles in degrees: Pd–C1 2.031(4), Pd–N3 2.137(2), Pd–C12 2.030(2), Pd–Cl1 2.342(1), C1–Pd–N3 80.1(1), C1–N1–C4–N3 −9.7(3).

**Figure 3:**
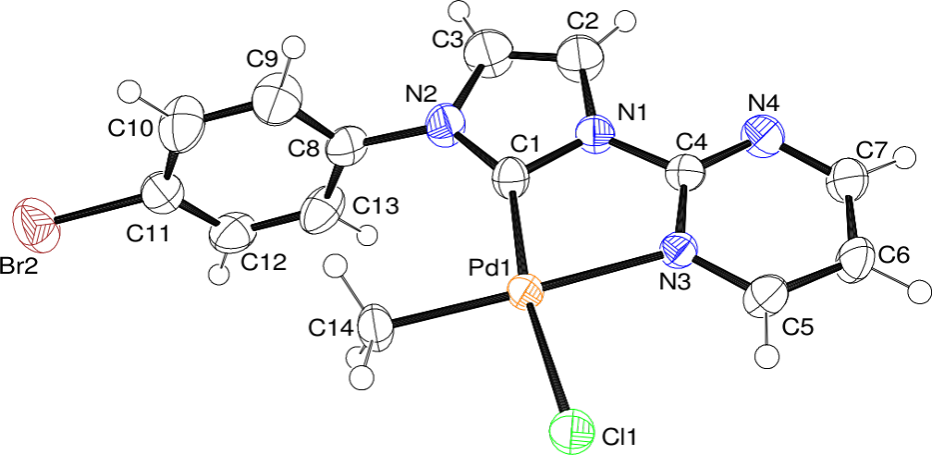
ORTEP [39] style plot of complex **12** in the solid state. Thermal ellipsoids are drawn at the 50% probability level. Selected bond lengths in angstrom and angles in degrees: Pd–C1 1.966(4), Pd–N3 2.145(3), Pd–C14 2.035(3), Pd–Cl1 2.341(1), C1–Pd–N3 79.0(1), C1–N1–C4–N3 −5.9(5).

For complexes **9** and **12** (Figure 2 and Figure 3) we were able to obtain solid state structures, which show the predicted geometry and confirm that the **B** isomers are formed.

It is interesting to note that the two structures show very similar bond lengths and angles with the exception of the palladium–carbene bond lengths (**9**: 2.03 Å; **12**: 1.97 Å), which might be an indication of the different donor character. The experimentally determined geometrical parameters are in good agreement with the computed results.

Interestingly, complexes **9**, **10** and **12** show only one discrete doublet for the *m*-pyrimidine proton signals in the ^1^H NMR spectrum in DMSO-*d*_6_ indicating that the pyrimidine ring might rotate at room temperature and therefore a weak coordination of the pyrimidine nitrogen atom to the palladium center. The broader signal in case of complex **11** indicates a stronger coordination of the nitrogen and a hindered rotation of the pyrimidine ring. Within some hours, complexes **9** and **12** decompose in DMSO-*d*_6_ by reductive *cis*-elimination [40] leading to imidazolium salts with the methyl group on the former carbene carbon atom and palladium black. The weaker coordination is most probably caused by the stronger *trans* effect of the methyl group. The decomposition is significantly slower in CDCl_3_, where two different signals for the *m*-pyrimidine protons are observed.

**Scheme 2:**
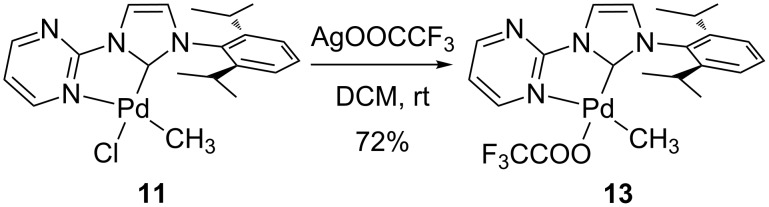
Synthesis of complex **13**.

**Figure 4:**
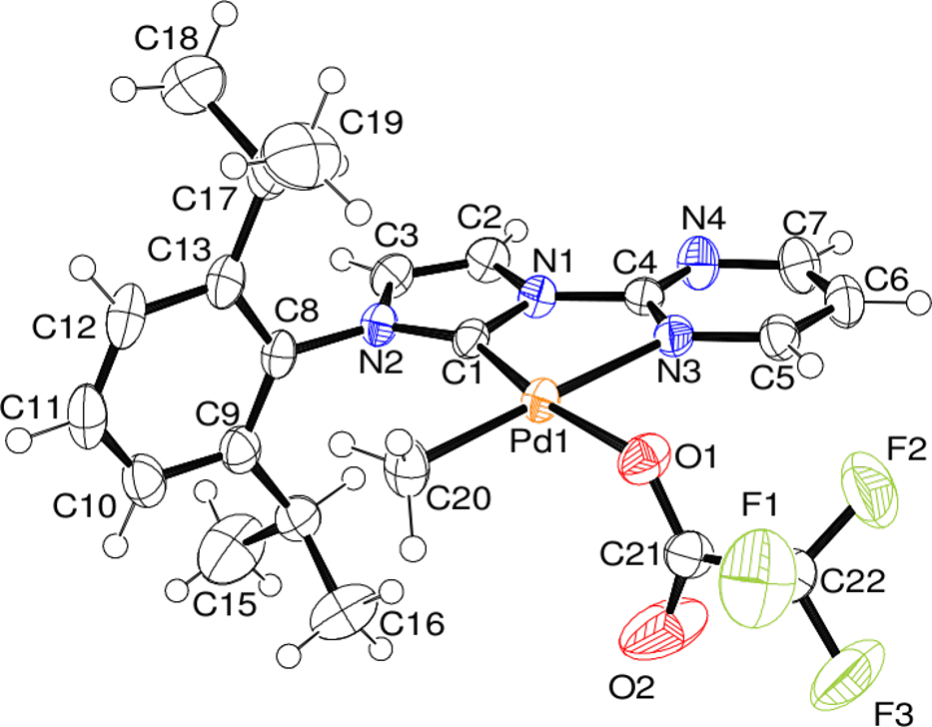
ORTEP [39] style plot of complex **13** in the solid state. Thermal ellipsoids are drawn at the 50% probability level. Selected bond lengths in angstrom and angles in degrees: Pd–C1 1.956(2), Pd–N3 2.151(2), Pd–C20 2.015(2), Pd–O1 2.098(2), C1–Pd–N3 79.6(1), C1–N1–C4–N3 −3.3(3).

We recently reported that the dihalogenato complexes are active catalysts of the methane CH activation in trifluoroacetic acid [34]. Under the reaction conditions it seems likely, that an exchange of the chloro against a trifluoroacetato ligands occurs [29], which is present in the solution in large excess. This potential intermediate of the catalytic cycle, the [(pym)^(NHC-R)Pd^II^(CH_3_)(CF_3_COO)]-complex **13** (with R = 2,6-diisopropylphenyl), could be synthesized by the reaction of complex **11** with silver trifluoroacetate (Scheme 2). We could also confirm the formation of the desired product by a solid state structure (Figure 4).

**Scheme 3:**
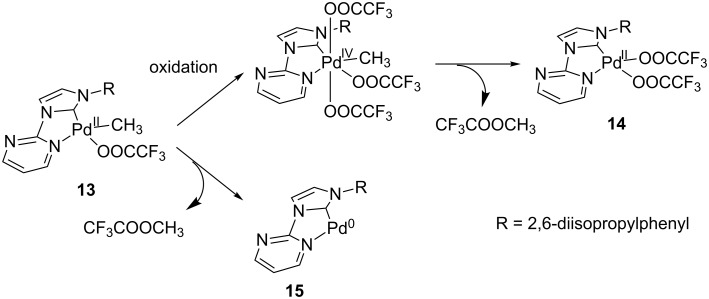
Possible pathways of methyl trifluoroacetate formation starting from complex **13**.

Typical reaction conditions of the catalytic CH activation are 30 atmospheres of methane under strongly oxidizing conditions (potassium peroxodisulfate, trifluoroacetic acid and its anhydride), where methane is converted into methyl trifluoroacetate catalyzed by a Pd(II) complex. To examine if a methylated Pd(II) complex like **13** can be an intermediate of this reaction, potassium peroxodisulfate and sodium trifluoroacetate were added to a solution of **13** in DMSO-*d*_6_ or CDCl_3_. Analyzing the solution we could confirm the formation of methyl trifluoroacetate and a ‘methyl free’ Pd(II) complex. This complex showed similar NMR spectra like the bistrifluoroacetate complex **14** (Scheme 3), which was prepared by the reaction of the corresponding dichloro complex [34] with silver trifluoroacetate for comparison (see experimental details). The result indicates that an oxidation/reductive elimination cycle took place (Scheme 3, upper pathway). The direct reductive elimination of methyl trifluoroacetate from complex **13** by heating complex **13** in DMSO-*d*_6_ up to 90 °C in the presence of sodium trifluoroacetate [30,34], yielding complex **15**, could not be observed (Scheme 3). This indicates that a P(II)/Pd(0)-mechanism is unfavorable. A more representative simulation of the reaction conditions of the catalytic methane activation by using trifluoroacetic acid as solvent was not possible as the protonation and dissociation of the methyl group occurs very quickly and quantitatively. The results obtained are in agreement with the computational results [41] that for this ligand system a Pd(II)/Pd(IV)-mechanism might be more favorable than a Pd(II)/Pd(0)-pathway for the formation of methyl trifluoroacetate from methylpalladium(II) complexes like **13** (Scheme 3).

**Scheme 4:**
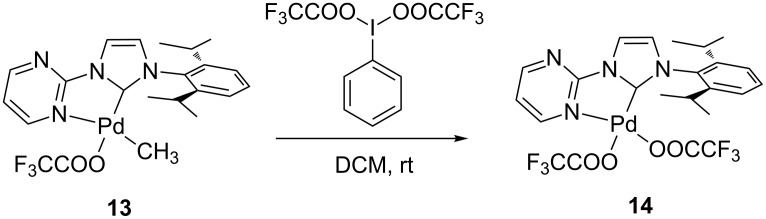
Synthesis of complex **14** by conversion of complex **13** with iodobenzene bistrifluoroacetate.

It has previously been shown that the oxidation of palladium(II) complexes is feasible [42]. Several papers reported the successful oxidation of Pd(II) complexes by hypervalent iodo reagents [43–48]. Sanford, Arnold and co-workers could also show, that the Pd(IV)(NHC) complexes prepared by this reaction tend to reductively eliminate at temperatures around −35 °C [49]. As we also consider a Pd(IV) intermediate, the corresponding complex [(pym)^(NHC-R)Pd^(IV)^(CH_3_)(CF_3_COO)_3_] was synthesized by reaction of **13** with iodobenzene bistrifluoroacetate at room temperature. We observed the formation of complex **14** as the only product (Scheme 4), while at a temperature of −78 °C no reaction was observed and complex **13** was reisolated from the reaction mixture.

We followed the reaction by NMR by mixing the reagents at −78 °C while measuring the ^1^H NMR spectra at −10 °C. After 5 minutes we observed a 1:1 mixture of complexes **13** and **14**. When the mixture warmed up to room temperature, the formation of complex **14** and methyl trifluoroacetate was detected. We believe that the reaction mechanism is similar to what was observed before by Sanford, Arnold and co-workers [49].

**Scheme 5:**
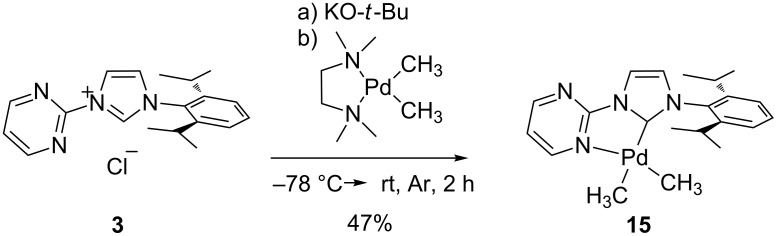
Synthesis of the [((pym)^(NHC-R))Pd^II^(CH_3_)_2_] complex **15**.

After we could successfully synthesize all the monomethyl complexes we were curious whether also the corresponding dimethyl complex is accessible. For this synthesis we decided to use the reaction of the free carbene with dimethyl(*N,N,N',N'-*tetramethyl-1,2-diaminoethylene)palladium(II), [(tmeda)Pd(CH_3_)_2_], an approach which is frequently used to synthesize dimethyl Pd complexes [50–53]. Deprotonation of the imidazolium salt **3** and subsequent conversion of the free carbene with [(tmeda)Pd(CH_3_)_2_] gave the desired dimethylpalladium(II) complex **15** (Scheme 5) in 47% yield.

## Conclusion

Methylpalladium(II) complexes are discussed as possible intermediates of the methane activation reaction. Herein, we present the synthesis of the potential intermediates ([((pym)^(NHC-R))Pd^II^(CH_3_)Cl] **9**–**12** with different substituents R (*t*-Bu, Cy, DIPP, 4-bromophenyl) of the corresponding catalytically active palladium dichloro complexes [(pym)^(NHC)Pd^II^(Cl)_2_] [34]. As the methane activation is carried out in a mixture of trifluoroacetic acid and its anhydride, another potential intermediate could be the [((pym)^(NHC-DIPP))Pd^II^(CH_3_)(CF_3_COO)] complex **13**. Formation of methyl trifluoroacetate and the bistrifluoroacetate complex **14** from this complex under oxidizing reaction conditions points to a Pd(II)/Pd(IV)-mechanism for the reductive elimination of the observed product methyl trifluoroacetate. The dimethyl complex [((pym)^(NHC-DIPP))Pd^II^(CH_3_)_2_] **15** could be synthesized to demonstrate the accessibility of these complexes as well.

## Experimental

^1^H and ^13^C NMR spectra were obtained on a Bruker AC 300 P (^1^H: 300.1 MHz, ^13^C: 75.5 MHz, ^19^F: 282.4 MHz) or a Bruker DRX 500 (^1^H: 500.1 MHz, ^13^C: 125.8 MHz, ^19^F: 470.3 MHz) spectrometer at 298 K. Elemental analyses were performed by the microanalytical laboratory of our institute using an EuroVektor Euro EA-300 Elemental Analyzer. Chemicals were supplied by Acros, Fluka and Aldrich and used as received; solvents were dried by standard procedures before use. Imidazolium salts **1**–**4** [34], silver complexes **5**–**8** [27], chloro(methyl)(cyclooctadiene)palladium(II) [54] and dimethyl-(*N*,*N*,*N'*,*N'*-tetramethyl-1,2-ethylendiamine)palladium(II) [38] were prepared according to literature procedures.

### Chloro(methyl)(1-(2-pyrimidyl)-3-(*tert*-butyl)imidazolin-2-ylidene)palladium(II) (**9**)

0.20 g (0.6 mmol) of the silver complex **5** and 0.16 g (0.6 mmol) chloro(methyl)(cyclooctadiene)palladium(II) are dissolved in 30 mL dichloromethane. Under exclusion of light in an argon atmosphere the reaction mixture is stirred for 9 h at room temperature. The resulting suspension is filtrated over a celite pad and concentrated in vacuo. The product is precipitated by the addition of diethyl ether, filtrated, washed with diethyl ether and dried in vacuo. The product is obtained as a yellow solid (0.16 g, 78%). Mp 165 °C (dec); ^1^H NMR (CDCl_3_, 300 MHz, ppm) δ 1.27 (s, 3H, Pd-C*H*_3_), 1.81 (s, 9H, C*H*_3_* t*-Bu), 7.26 (d, *J =* 2.2 Hz, 1H, NC*H*), 7.43 (t, *J =* 5.1 Hz, 1H, *p-H* pym), 7.91 (d, *J* = 2.2 Hz, 1H, NC*H*), 9.21 (bs, 2H, *m-H* pym); ^13^C NMR (DMSO-*d*_6_, 75.5 MHz, ppm) δ −1.5 (Pd-*C*H_3_), 31.2 (*C*H_3_), 59.9 (*ipso-C t*-Bu), 116.0 (*p-C*H pym), 120.1 (N*C*H), 120.9 (N*C*H), 156.4 (*ipso-C* pym), 159.4 (*m-C*H pym), 170.0 (carbene-*C*); anal. calcd for C_12_H_17_ClN_4_Pd·0.2CH_2_Cl_2_: C, 39.34%; H, 4.63%; N, 14.80%; found: C, 39.68%; H, 4.46%; N, 15.32%.

### Chloro(methyl)(1-(2-pyrimidyl)-3-(cyclohexyl)imidazolin-2-ylidene)palladium(II) (**10**)

0.10 g (0.3 mmol) of the silver complex **6** and 0.08 g (0.3 mmol) chloro(methyl)(cyclooctadiene)palladium(II) are dissolved in 30 mL dichloromethane. Under exclusion of light in an argon atmosphere the reaction mixture is stirred for 9 h at room temperature. The resulting suspension is filtrated over a celite pad and concentrated in vacuo. The product is precipitated by the addition of diethyl ether, filtrated, washed with diethyl ether and dried in vacuo. The product is obtained as a yellow solid (0.10 g, 97%). Mp 167 °C (dec); ^1^H NMR (DMSO-*d*_6_, 300 MHz, ppm) δ 0.87 (s, 3H, Pd-C*H*_3_), 1.23–1.96 (2m, 10H, cyc), 4.45 (m, 1H, NC*H* cyc), 7.70 (t, *J =* 5.1 Hz, 1H, *p-H* pym), 7.80 (d, *J* = 2.3 Hz, 1H, NC*H*), 8.16 (d, *J* = 2.3 Hz, 1H, NC*H*), 9.07 (d, 5.1 Hz, 2H, *m-H* pym); ^13^C NMR (DMSO-*d*_6_, 75.5 MHz, ppm) δ −11.6 (Pd-*C*H_3_), 24.4 (*p-C*H_2_ cyc), 25.0 (*m-C*H_2_ cyc), 32.5 (*o-C*H_2_ cyc), 57.7 (N*C*H cyc), 117.2 (*p-C*H pym), 120.2 (N*C*H), 120.7 (N*C*H), 155.4 (*ipso-C* pym), 158.4 (*m-C*H pym), 159.6 (*m-C*H pym), 167.3 (carbene-*C*); anal. calcd for C_14_H_19_ClN_4_Pd·0.1AgCl: C, 42.09%; H, 4.79%; N, 14.02%; found: C, 42.27%; H, 4.71%; N, 13.70%.

### Chloro(methyl)(1-(2-pyrimidyl)-3-(2,6-diisopropylphenyl)imidazolin-2-ylidene)palladium(II) (**11**)

0.04 g (0.1 mmol) of the silver complex **7** and 0.03 g (0.1 mmol) chloro(methyl)(cyclooctadiene)palladium(II) are dissolved in 30 mL dichloromethane. Under exclusion of light in an argon atmosphere the reaction mixture is stirred for 9 h at room temperature. The resulting suspension is filtrated over a celite pad and concentrated in vacuo. The product is precipitated by the addition of diethyl ether, filtrated, washed with diethyl ether and dried in vacuo. The product is obtained as a white solid (0.04 g, 84%). Mp 158 °C (dec); ^1^H NMR (DMSO-*d*_6_, 300 MHz, ppm) δ 0.04 (s, 3H, Pd-C*H*_3_), 1.12 (d, *J =* 6.8 Hz, 6H, C*H*_3_), 1.22 (d, *J* = 6.8 Hz, 6H, C*H*_3_), 2.59 (m, 2H, C*H*), 7.36 (d, *J* = 7.7 Hz, 2H, *o-H* ph), 7.53 (t, *J* = 7.7 Hz, 1H, *p-H* ph), 7.75 (t, *J* = 5.1 Hz, 1H, *p-H* pym), 7.77 (d, *J* = 2.3 Hz, 1H, NC*H*), 8.33 (d, *J* = 2.2 Hz, 1H, NC*H*), 9.09 (bs, 2H, *m-C*H pym); ^13^C NMR (DMSO-*d*_6_, 75.5 MHz, ppm) δ −10.7 (Pd-*C*H_3_), 22.8, 24.3 (*C*H_3_ iPr), 27.9 (*C*H), 117.1 (*p-C*H pym), 120.7 (N*C*H), 123.7 (*m-C*H ph), 126.8 (N*C*H), 130.3 (*p-C*H ph), 134.4 (*ipso-C* ph), 144.5 (*o-C* ph), 155.3 (*ipso-C* pym), 156.7 (*m-C*H pym), 160.4 (*m-C*H pym), 170.7 (carbene-*C*); anal. calcd for C_20_H_25_ClN_4_Pd: C, 51.85%; H, 5.44%; N, 12.09%; found: C, 51.66%; H, 5.73%; N 11.65%.

### Chloro(methyl)(1-(2-pyrimidyl)-3-(4-bromophenyl)imidazolin-2-ylidene)palladium(II) (**12**)

0.05 g (0.1 mmol) of silver complex **8** and 0.03 g (0.1 mmol) chloro(methyl)(cyclooctadiene)palladium(II) are dissolved in 30 mL acetonitrile. Under exclusion of light the reaction mixture is stirred in an argon atmosphere for 72 h at room temperature. The resulting suspension is filtrated over a celite pad and the solvent is removed in vacuo. The product is recrystallized from acetonitrile and dried in vacuo. The product is obtained as a white solid 0.02 g (48%). Mp 202 °C (dec); ^1^H NMR (CDCl_3_, 300 MHz, ppm) δ 0.04 (s, 3H, Pd-C*H*_3_), 7.01 (d, *J =* 2.2 Hz, 1H, NC*H*), 7.37 (d, *J* = 8.7 Hz, 2H, *o-H* ph), 7.48 (t, *J* = 5.1 Hz, 1H, *p-H* pym), 7.66 (d, *J* = 8.7 Hz, 2H, *m-H* ph), 7.99 (d, *J* = 2.2 Hz, 1H, NC*H*), 8.83 (bs, 1H, *m-H* pym), 9.39 (bs, 1H, *m-H* pym); ^13^C NMR (CDCl_3_, 75.5 MHz, ppm) δ −7.4 (Pd-*C*H_3_), 116.8 (*p-C*H pym), 120.2 (N*C*H), 124.2 (*p*-C ph), 124.8 (N*C*H), 125.9 (*ipso-C* ph), 128.0 (*o-C*H ph), 132.8 (*m-C*H ph), 154.9 (*m-C*H pym), 155.6 (*ipso-C* pym); anal. calcd for C_14_H_12_BrClN_4_Pd·0.1AgCl: C, 35.60%; H, 2.56%; N, 11.86%; found: C, 35.98%; H, 2.14%; N, 12.16%.

### Methyl(trifluoroacetato)(1-(2-pyrimidyl)-3-(2,6-diisopropylphenyl)imidazolin-2-ylidene)palladium(II) (**13**)

0.10 g (0.2 mmol) of **11** and 0.05 g (0.2 mmol) silver trifluoroacetate are dissolved in 100 mL dichloromethane. Under exclusion of light the reaction mixture is stirred under an argon atmosphere for 9 h at room temperature. The resulting suspension is filtrated over a celite pad and concentrated in vacuo. The product is precipitated by the addition of diethyl ether, filtrated, washed with diethyl ether and dried in vacuo. The product is obtained as a white solid (0.08 g, 82%). Mp 232 °C (dec); ^1^H NMR (DMSO-*d*_6_, 300 MHz, ppm) δ 0.09 (s, 3H, Pd-C*H*_3_), 1.13 (d, *J* = 6.8 Hz, 6H, C*H*_3_), 1.23 (d, *J* = 6.8 Hz, 6H, C*H*_3_), 2.55 (sept, *J* = 6.8 Hz, 2H, C*H*), 7.39 (d, *J* = 7.8 Hz, 2H, *o-H* ph), 7.56 (t, *J* = 7.8 Hz, 1H, *p-H* ph), 7.79 (t, *J =* 5.2 Hz, 1H, *p-H* pym), 7.89 (d, *J =* 2.1 Hz, 1H, NC*H*), 8.43 (d, *J =* 2.1 Hz, 1H, NC*H*), 8.99 (bs, 2H, *m-C*H pym); ^13^C NMR (DMSO-*d*_6_, 75.5 MHz, ppm) δ −6.9 (Pd-*C*H_3_), 23.0 (*C*H_3_ iPr), 24.3 (*C*H_3_ iPr), 28.0 (*C*H), 118.1 (*p-C*H pym), 121.1 (N*C*H), 124.0 (*m-C*H ph), 127.4 (N*C*H), 130.7 (*p-C*H ph), 134.2 (*ipso-C* ph), 144.6 (*o-C* ph), 155.0 (*ipso-C* pym), *m-C*H pym and carben-*C* not observed in DMSO-*d*_6_.

### Bis(trifluoroacetato)(1-(2-pyrimidyl)-3-(2,6-diisopropylphenyl)imidazolin-2-ylidene)palladium(II) (**14**)

Synthetic pathway 1: 0.05 g (0.1 mmol) of **13** and 0.08 g (0.2 mmol) iodobenzene bis(trifluoroacetate) are dissolved in 10 mL dichloromethane. The reaction mixture is stirred for 12 h at room temperature. The solvent is removed in vacuo, the resulting solid is washed with THF. The product is obtained as a white solid (quant.). Mp 259 °C (dec).

Synthetic pathway 2, starting from dichloro(1-(2-pyrimidyl)-3-(2,6-diisopropylphenyl)imidazolin-2-ylidene)palladium(II) [34]: 0.03 g (0.1 mmol) dichloro(1-(2-pyrimidyl)-3-(2,6-diisopropylphenyl)imidazolin-2-ylidene)palladium(II) and 0.05 g (0.2 mmol) silver trifluoroacetate are dissolved in 10 mL dichloromethane at 0 °C. The reaction mixture is allowed to warm up to room temperature and stirred for another 4 h. The resulting suspension is filtrated over a celite pad, the solvent is removed in vacuo and the resulting solid washed with THF. The product is obtained as a white solid (quant.). Mp 259 °C (dec); ^1^H NMR (DMSO-*d*_6_, 300 MHz, ppm) δ 1.08 (d, *J* = 6.6 Hz, 6H, C*H*_3_), 1.28 (d, *J* = 6.6 Hz, 6H, C*H*_3_), 2.62 (sept, *J* = 6.6 Hz, 2H, C*H*), 7.29 (d, *J* = 7.8 Hz, 2H, *o-H* ph), 7.46 (t, *J =* 7.7 Hz, 1H, *p-H* ph), 7.79 (t, *J =* 5.2 Hz, 1H, *p-H* pym), 7.93 (s, 1H, NC*H*), 8.50 (d, *J =* 1.3 Hz, 1H, NC*H*), 8.68 (m, 1H, *m-C*H pym), 9.19 (m, 1H, *m-C*H pym); ^13^C NMR (DMSO-*d*_6_, 75.5 MHz, ppm) δ 22.4 (*C*H_3_ iPr), 24.8 (*C*H_3_ iPr), 27.9 (*C*H iPr), 118.1 (*p-C*H pym), 120.4 (N*C*H), 123.7 (*m-C*H ph), 127.2 (N*C*H), 130.4 (*p-C*H ph), 131.7 (*ipso-C* ph), 144.3 (*o-C* ph), 156.2 (*ipso-C* pym), 162.1 (*m-C*H pym), carbene-*C* not observed; ^19^F NMR (DMSO-*d*_6_, 75.5 MHz, ppm) δ −72.54 (CF_3_), −73.47 (CF_3_); anal. calcd for C_23_H_22_F_6_N_4_O_4_Pd: C, 43.24%; H, 3.47%; N, 8.77%; found: C, 42.73%; H, 3.67%; N, 8.38%.

### Dimethyl(1-(2-pyrimidyl)-3-(2,6-diisopropylphenyl)imidazolin-2-ylidene)palladium(II) (**15**)

0.18 g (0.6 mmol) of the imidazolium salt **3** and 0.07 g (0.6 mmol) potassium *tert*-butanolat are suspended in dry THF at −78 °C under an atmosphere of argon. Over a period of 2 h, the reaction mixture is allowed to warm up to 0 °C. Afterwards the solution is again cooled to −78 °C. A cold solution (−78 °C) of 0.10 g (0.4 mmol) dimethyl(*N,N,N',N'-*tetramethyl-1,2-diaminoethylene)palladium(II) in 20 mL THF is added. The reaction mixture is allowed to warm up to 0 °C over a period of 2 h, the solvent is removed in vacuo and the resulting precipitate is extracted with dry toluene (2 × 10 mL). The solvent is concentrated in vacuo again and the product is precipitated by the addition of 20 mL pentane. The solid is filtrated, washed with cold pentane and dried in vacuo. Yield: 0.13 g (47%). ^1^H NMR (C_6_D_6_, 300 MHz, ppm) δ 0.68 (s, 3H, Pd-C*H*_3_), 0.94 (s, 3H, Pd-C*H*_3_), 1.04 (d, *J* = 6.9 Hz, 6H, C*H*_3_), 1.41 (d, *J* = 6.9 Hz, 6H, C*H*_3_), 2.85 (sept, *J* = 6.8 Hz, 2H, C*H*), 5.94 (t, *J* = 5.9 Hz, 1H, *p-H* pym), 6.17 (d, *J =* 2.0 Hz, 1H, NC*H*), 7.12 (d, *J* = 7.6 Hz, 2H, *m-H* ph), 7.26 (t, *J =* 7.3 Hz, 1H, *p-H* ph), 7.49 (d, *J =* 2.0 Hz, 1H, NC*H*), 7.67 (m, 1H, *m-C*H pym), 8.53 (m, 1H, *m-C*H pym); ^13^C NMR (C_6_D_6_, 75.5 MHz, ppm) δ = −10.9 (Pd-*C*H_3_), 1.7 (Pd-*C*H_3_), 23.9 (*C*H_3_ iPr), 24.3 (*C*H_3_ iPr), 28.9 (*C*H iPr), 115.3 (*p-C*H pym), 118.9 (N*C*H), 124.0 (*m-C*H ph), 124.5 (N*C*H), 130.4 (*p-C*H ph), 135.8 (*ipso-C* ph), 145.6 (*o-C* ph), 155.3 (*ipso-C* pym), 156.1 (*m-C*H pym), 157.0 (*m-C*H pym), 194.7 (carbene-*C*). ESIMS: 444.1 [M + H]^+^.

### Computational details

All calculations were performed with the Gaussian 09 software [55]. The geometries were optimized using the density functional hybrid model B3LYP [56–60] together with the split valence double-ζ 6-31G(d) [61–66] and triple-ζ 6-311+G(d,p) [67–74] basis sets. All reported structures were verified as true minima by the absence of negative eigenvalues in the vibrational frequency analysis. All energies reported are Gibbs free energies at standard conditions (*T* = 298 K, *p* = 1 atm) using unscaled frequencies. For visualization GaussView [75] was used.

### Solid-state structure determination of **9**, **12** and **13**

Preliminary examination and data collection were carried out on an area detecting system (Kappa-CCD; Nonius) at the window of a sealed X-ray tube (Nonius, FR590) and graphite monochromated Mo Kα radiation (λ = 0.72073 Å). The reflections were integrated. Raw data were corrected for Lorentz and polarization and, arising from the scaling procedure, for latent decay. An absorption correction was applied using SADABS [76]. After merging, the independent reflections were all used to refine the structures, which were solved by a combination of direct methods and difference Fourier synthesis. All non-hydrogen atoms were refined with anisotropic displacement parameters. All hydrogen atoms were placed in calculated positions and refined using the riding model. Full-matrix least-squares refinements were carried out by minimizing Σw(F_o_^2^ − F_c_^2^)^2^. Details of the structure determinations are given in Table S1 (Supporting Information File 1). Neutral atom scattering factors for all atoms and anomalous dispersion corrections for the non-hydrogen atoms were taken from the International Tables for Crystallography [77]. All calculations were performed with the SHELXL-97 [78] package and the programs COLLECT [79], DIRAX [80], EVALCCD [81], SIR-92 [82], SADABS [76], ORTEP III [83] and PLATON [84].

## Supporting Information

Supplementary crystallographic data can be obtained free of charge from The Cambridge Crystallographic Data Centre via http://www.ccdc.cam.ac.uk/data_request/cif.

File 1 Details of the DFT calculations and additional crystallographic data for **9** (CCDC 1484282), **12** (CCDC 1484281) and **13** (CCDC 1484283).

File 2 CIF file for compound **9**.

File 3 CIF file for compound **12**.

File 4 CIF file for compound **13**.

## References

[1] Tsuji J (2015). Tetrahedron.

[2] Johansson Seechurn C C C, Kitching M O, Colacot T J, Snieckus V (2012). Angew Chem, Int Ed.

[3] Hartwig J F (2016). J Am Chem Soc.

[4] Campbell A N, Stahl S S (2012). Acc Chem Res.

[5] Labinger J A (2012). Alkane Functionalization via Electrophilic Activation. Alkane C-H Activation by Single-Site Metal Catalysis.

[6] Lyons T W, Sanford M S (2010). Chem Rev.

[7] Arndtsen B A, Bergman R G, Mobley T A, Peterson T H (1995). Acc Chem Res.

[8] Janowicz A H, Bergman R G (1982). J Am Chem Soc.

[9] Janowicz A H, Bergman R G (1983). J Am Chem Soc.

[10] Hoyano J K, Graham W A G (1982). J Am Chem Soc.

[11] Hoyano J K, McMaster A D, Graham W A G (1983). J Am Chem Soc.

[12] Shteinman A A (2015). J Organomet Chem.

[13] Crabtree R H (2015). J Organomet Chem.

[14] Shestakov A F, Goldshleger N F (2015). J Organomet Chem.

[15] Stahl S S, Labinger J A, Bercaw J E (1998). Angew Chem, Int Ed.

[16] Gol'dshleger N F, Es'kova V V, Shilov A E, Shteinman A A (1972). Zh Fiz Khim.

[17] Gol'dshleger N F, Tyabin M B, Shilov A E, Shteinman A A (1969). Zh Fiz Khim.

[18] Periana R A, Taube D J, Gamble S, Taube H, Satoh T, Fujii H (1998). Science.

[19] Kua J, Xu X, Periana R A, Goddard W A (2002). Organometallics.

[20] Ahlquist M, Nielsen R J, Periana R A, Goddard W A (2009). J Am Chem Soc.

[21] Sen A (1991). Platinum Met Rev.

[22] Balcer S (2015). Pol J Chem Technol.

[23] Caballero A, Pérez P J (2013). Chem Soc Rev.

[24] Munz D, Strassner T (2015). Inorg Chem.

[25] Munz D, Strassner T (2014). Top Catal.

[26] Munz D, Meyer D, Strassner T (2013). Organometallics.

[27] Ahrens S, Zeller A, Taige M, Strassner T (2006). Organometallics.

[28] Strassner T, Ahrens S, Zeller A (2007). N-heterocyclische Carbenkomplexe des Platins und des Palladiums, deren Herstellung und Verwendung zur partiellen Oxidation von Kohlenwasserstoffen.

[29] Strassner T, Muehlhofer M, Zeller A, Herdtweck E, Herrmann W A (2004). J Organomet Chem.

[30] Muehlhofer M, Strassner T, Herrmann W A (2002). Angew Chem, Int Ed.

[31] Ahrens S, Strassner T (2006). Inorg Chim Acta.

[32] Maletz G, Schmidt F, Reimer A, Strassner T, Muehlhofer M, Mihalios D, Herrmann W (2006). Katalysator und Verfahren zur partiellen Oxidation von Alkanen.

[33] Brendel M, Engelke R, Desai V G, Rominger F, Hofmann P (2015). Organometallics.

[34] Meyer D, Taige M A, Zeller A, Hohlfeld K, Ahrens S, Strassner T (2009). Organometallics.

[35] Meyer D, Zeller A, Strassner T (2012). J Organomet Chem.

[36] Sperger T, Sanhueza I A, Kalvet I, Schoenebeck F (2015). Chem Rev.

[37] Meyer D, Strassner T (2015). J Organomet Chem.

[38] Byers P K, Canty A J (1990). Organometallics.

[39] Farrugia L J (1997). J Appl Crystallogr.

[40] Magill A M, McGuinness D S, Cavell K J, Britovsek G J P, Gibson V C, White A J P, Williams D J, White A H, Skelton B W (2001). J Organomet Chem.

[R41] 41Meyer, D. Azoliumsalze als NHC-Precursoren fur Katalysatoren (Pd, Pt) zur Methanaktivierung sowie als ionische Flussigkeiten – Synthese, DFT Rechnungen und mechanistische Studien. Dissertation, TU Dresden, 2011.

[42] McCall A S, Wang H, Desper J M, Kraft S (2011). J Am Chem Soc.

[43] Pérez-Temprano M H, Racowski J M, Kampf J W, Sanford M S (2014). J Am Chem Soc.

[44] Racowski J M, Ball N D, Sanford M S (2011). J Am Chem Soc.

[45] Racowski J M, Dick A R, Sanford M S (2009). J Am Chem Soc.

[46] Powers D C, Ritter T (2009). Nat Chem.

[47] Whitfield S R, Sanford M S (2007). J Am Chem Soc.

[48] Dick A R, Kampf J W, Sanford M S (2005). J Am Chem Soc.

[49] Arnold P L, Sanford M S, Pearson S M (2009). J Am Chem Soc.

[50] Danopoulos A A, Tsoureas N, Macgregor S A, Smith C (2007). Organometallics.

[51] Subramanium S S, Slaughter L M (2009). Dalton Trans.

[52] Douthwaite R E, Green M L H, Silcock P J, Gomes P T (2002). J Chem Soc, Dalton Trans.

[53] Tsoureas N, Danopoulos A A, Tulloch A A D, Light M E (2003). Organometallics.

[54] Salo E V, Guan Z (2003). Organometallics.

[55] (2009). Gaussian 09.

[56] Stephens P J, Devlin F J, Chabalowski C F, Frisch M J (1994). J Phys Chem.

[57] Miehlich B, Savin A, Stoll H, Preuss H (1989). Chem Phys Lett.

[58] Becke A D (1988). Phys Rev A.

[59] Vosko S H, Wilk L, Nusair M (1980). Can J Phys.

[60] Lee C, Yang W, Parr R G (1988). Phys Rev B.

[61] Rassolov V A, Pople J A, Ratner M A, Windus T L (1998). J Chem Phys.

[62] Hehre W J, Ditchfield R, Pople J A (1972). J Chem Phys.

[63] Hariharan P C, Pople J A (1974). Mol Phys.

[64] Hariharan P C, Pople J A (1972). Chem Phys Lett.

[65] Hariharan P C, Pople J A (1973). Theor Chim Acta.

[66] Ditchfield R, Hehre W J, Pople J A (1971). J Chem Phys.

[67] Binning R C, Curtiss L A (1990). J Comput Chem.

[68] Clark T, Chandrasekhar J, Spitznagel G W, Von Ragué Schleyer P (1983). J Comput Chem.

[69] Frisch M J, Pople J A, Binkley J S (1984). J Chem Phys.

[70] Hay P J (1977). J Chem Phys.

[71] Krishnan R, Binkley J S, Seeger R, Pople J A (1980). J Chem Phys.

[72] McGrath M P, Radom L (1991). J Chem Phys.

[73] Raghavachari K, Trucks G W (1989). J Chem Phys.

[74] Wachters A J H (1970). J Chem Phys.

[R75] 75Dennington, R. I.; Keith, T.; Millam, J. M.; Eppinnett, W.; Hovell, W. L.; Gilliland, R. *GaussView*, 3.09; Gaussian, Inc.: Wallingford, CT, 2003.

[R76] 76Sheldrick, G. M., *SADABS, Version 2.10.* University of Goettingen, Goettingen, Germany, 2003.

[77] Wilson A J C (1992). International Tables for Crystallography.

[R78] 78Sheldrick, G. M., *SHELXL-97, Program for the Refinement of Structures.* University of Goettingen, Goettingen, Germany, 1997.

[R79] 79Nonius, *Data Collection Software for Nonius-kappa CCD devices.* Delft, The Netherlands, 1997–2000.

[80] Duisenberg A J M (1992). J Appl Crystallogr.

[81] Duisenberg A J M, Hooft R W W, Schreurs A M M, Kroon J (2000). J Appl Crystallogr.

[82] Altomare A, Cascarano G, Giacovazzo C, Guagliardi A, Burla M C, Polidori G, Camalli M (1994). J Appl Crystallogr.

[83] Burnett M N, Johnson C K (1996). ORTEPIII.

[84] Spek A L (2001). PLATON.

