# Supplementary material for: Methylpalladium complexes with pyrimidine-functionalized N-heterocyclic carbene ligands
Source: Beilstein J Org Chem. 2016 Jul 21;12:1557–65. doi: 10.3762/bjoc.12.150 (PMC4979653; doi:10.3762/bjoc.12.150)
Supplement: File 1 — Details of the DFT calculations and additional crystallographic data for 9 (CCDC 1484282), 12 (CCDC 1484281) and 13 (CCDC 1484283). [file Beilstein_J_Org_Chem-12-1557-s001.pdf]

Supporting Information

for

Methylpalladium complexes with pyrimidine-functionalized N-heterocyclic carbene ligands

Dirk Meyer and Thomas Strassner*[§]

Address: Physikalische Organische Chemie, TU Dresden, Bergstraße 66, 01062 Dresden

Email: Thomas Strassner - thomas.strassner@chemie.tu-dresden.de,

*Corresponding author

[§]Tel.: ++49 351 4633 8571; Fax: ++49 351 4633 9679

Details of the DFT calculations and additional crystallographic data for 9 (CCDC 1484282), 12 (CCDC 1484281) and 13 (CCDC 1484283)

Content:

Details of the solid state structure determination S2

Summary of calculated energies S3

XYZ coordinates S4

Details of the solid state structure determination

Table S1. Crystal data and crystallographic details for complexes **9, 12** and **13**.

Complex	**9**	**12**	**13**
formula	$C_{12}H_{17}ClN_4Pd$	$C_{14}H_{12}BrClN_4Pd$	$C_{22}H_{25}F_3N_4O_2Pd$
fw [g/mol]	359.15	458.05	540.86
T [K]	198(2)	198(2)	198(2)
wavelength [Å]	0.71073	0.71073	0.71073
crystal system	Monoclinic	Monoclinic	Monoclinic
space group	P 21/c	P 21/c	P 21/c
a [Å]	7.9910(6)	11.0550(8)	9.2900(6)
b [Å]	19.3720(13)	8.0500(5)	20.254(2)
c [Å]	11.0910(9)	17.9200(10)	15.2870(6)
α [°]	90	90	90
β [°]	127.399(8)	102.069(6)	127.010(4)
γ [°]	90	90	90
U [$Å^3$]	1363.95(18)	1559.50(17)	2296.9(3)
Z	4	4	4
D_{calc} [Mg/m^3]	1.749	1.951	1.564
μ(MoKα) [mm^{-1}]	1.544	3.920	0.858
crystal size [mm^3]	0.31x0.14x 0.10	0.20x0.13x 0.05	0.52x0.47x 0.20
F(000)	720	888	1096
refl. collected	22194	18071	27751
independent refl.	2270 [R_{int}=0.0356]	3175 [R_{int}=0.0709]	4684 [R_{int}=0.0250]
Goof on F^2	1.146	1.098	1.508
R_1 [I>2σ(I)]	0.0203	0.0311	0.0263
wR_2	0.0513	0.0605	0.0769
data/restr/param	2779/0/167	3175/0/191	4684/0/289

Summary of energies (B3LYP/6-31G(d)):

	Isomer **A** 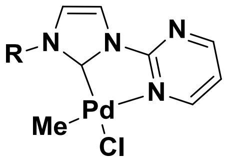			Isomer **B** 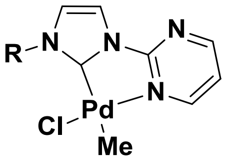		
	HF	ΔH	ΔG	HF	ΔH	ΔG
**9**	-1273,49605	-1273,19336	-1273,26022	-1273,48864	-1273,18667	-1273,25284
**10**	-1350,92997	-1350,58853	-1350,65865	-1350,91727	-1350,57643	-1350,64684
**11**	-1583,17572	-1582,72798	-1582,81425	-1583,15884	-1582,71173	-1582,79749
**12**	-3920,78911	-3920,52885	-3920,60074	-3920,77309	-3920,51318	-3920,58448

Summary of energies (B3LYP/6-311+G(d,p)):

	Isomer **A** 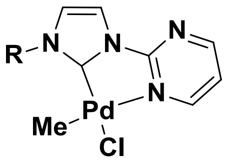			Isomer **B** 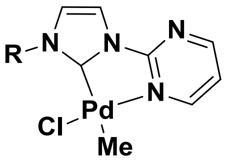		
	HF	ΔH	ΔG	HF	ΔH	ΔG
**9**	-1273,72000	-1273,41991	-1273,48724	-1273,71234	-1273,41317	-1273,47970
**10**	-1351,17204	-1350,83348	-1350,90402	-1351,15939	-1350,82146	-1350,89235
**11**	-1583,48032	-1583,03646	-1583,12387	-1583,46394	-1583,02070	-1583,10679
**12**	-3921,07408	-3920,81579	-3920,88793	-3921,05816	-3920,80025	-3920,87193

XYZ coordinates of calculated structures (B3LYP/6-31G(d))

9, Isomer **A**:

6	-3.355779	-0.598598	1.342131
6	-4.563522	1.072345	-0.060853
6	-2.089849	2.406821	-0.030937
6	-0.814430	2.843804	-0.027390
6	3.267271	0.423011	0.015652
6	4.031316	1.588702	0.011410
6	-3.319801	0.168618	0.007893
6	1.373210	1.698456	-0.000972
6	3.340240	2.798794	0.000575
6	-0.789392	0.532801	0.000669
6	-0.827447	-2.759048	-0.014736
6	-3.318767	-0.737396	-1.235843
1	-3.490016	0.101879	2.174458
1	-2.435388	-1.155546	1.513534
1	-4.197348	-1.299347	1.346623
1	-4.643045	1.741260	0.802805
1	-0.376436	-3.500257	0.647335
1	-3.002734	2.974403	-0.045225
1	-0.380647	3.829258	-0.037494
1	-5.446969	0.427033	-0.054731
1	3.679683	-0.583212	0.022154
1	5.114468	1.551746	0.016011
1	3.866611	3.750634	-0.003256
1	-1.848068	-2.546922	0.296408
1	-4.597313	1.665275	-0.981258
1	-0.813701	-3.136189	-1.043153
1	-4.174154	-1.420166	-1.196006
1	-2.406576	-1.325940	-1.311613
1	-3.410973	-0.127392	-2.141791
17	2.222462	-2.754907	-0.016882
7	1.929821	0.484971	0.009858
7	-2.068534	1.007213	-0.014602
7	-0.029754	1.698789	-0.008164
7	2.001636	2.868392	-0.006044
46	0.442397	-1.155620	0.008028

9, Isomer **B**:

6	3.263557	-0.073153	-1.273910
6	4.177309	1.883407	0.000230
6	1.499982	2.776025	0.000102
6	0.164560	2.970957	0.000020
6	-3.392297	-0.189721	-0.000070
6	-4.358976	0.810008	-0.000327
6	3.113838	0.773379	0.000130
6	-1.754995	1.406442	-0.000098
6	-3.903215	2.126042	-0.000437
6	0.574812	0.696357	0.000024
6	3.263469	-0.073264	1.274105
1	3.126625	0.548172	-2.167002
1	2.547887	-0.897232	-1.287506

1	4.271561	-0.500944	-1.306159
1	4.121541	2.516014	-0.893022
1	2.292096	3.503614	0.000173
1	-0.442076	3.860465	-0.000038
1	5.161479	1.406344	0.000202
1	-3.646887	-1.243203	0.000047
1	-5.415201	0.567774	-0.000438
1	-4.592476	2.967274	-0.000655
1	4.121518	2.515864	0.893586
1	4.271448	-0.501112	1.306344
1	2.547755	-0.897301	1.287622
1	3.126550	0.547996	2.167245
7	-2.080449	0.096349	0.000063
7	1.736463	1.394465	0.000100
7	-0.389787	1.692983	-0.000028
7	-2.601093	2.430266	-0.000339
46	-0.444619	-1.256786	0.000036
6	-1.711413	-2.875011	0.000301
1	-2.331986	-2.810724	0.904430
1	-2.332090	-2.810975	-0.903774
1	-1.153236	-3.810013	0.000398
17	1.323662	-2.813947	-0.000123

10, Isomer **A**:

6	-1.385120	2.506858	-0.000929
6	-0.088441	2.886637	-0.001029
6	3.827362	0.165633	0.000768
6	2.046128	1.598227	-0.000255
6	-0.170100	0.582828	-0.000301
6	4.686319	1.264098	0.000715
6	4.098193	2.527644	0.000112
1	-2.283762	3.102393	-0.001176
1	0.388368	3.852510	-0.001318
1	4.153949	-0.871898	0.001164
1	5.762731	1.137846	0.001126
1	4.703036	3.431637	0.000069
46	0.864570	-1.167101	-0.000041
7	-1.418060	1.107626	-0.000436
7	0.647928	1.706081	-0.000656
7	2.501077	0.339135	0.000293
7	2.769554	2.711000	-0.000367
6	-0.691258	-2.486414	-0.000831
1	-1.297952	-2.347774	0.901038
1	-1.298464	-2.346355	-0.902128
1	-0.250395	-3.483902	-0.001708
17	2.440973	-2.965791	0.000419
6	-2.667329	0.323122	0.000250
6	-3.491322	0.583547	1.272780
6	-3.491165	0.580903	-1.272935
1	-2.343314	-0.715960	0.001406
6	-4.770590	-0.269423	1.269617
1	-3.763624	1.647649	1.327736
1	-2.879668	0.363002	2.155621

6	-4.770487	-0.271971	-1.268169
1	-3.763302	1.644936	-1.330112
1	-2.879404	0.358468	-2.155224
6	-5.604822	-0.035113	0.000446
1	-5.362999	-0.046656	2.165112
1	-4.494665	-1.331433	1.334040
1	-5.362801	-0.050954	-2.164160
1	-4.494586	-1.334111	-1.330475
1	-6.485288	-0.689057	0.001067
1	-5.982326	0.998402	-0.000606

10, Isomer **B**:

6	1.049040	-2.656210	-0.001202
6	-0.277702	-2.921386	-0.000989
6	-3.913221	0.188449	0.001009
6	-2.270396	-1.406326	0.000092
6	0.014522	-0.635727	-0.000275
6	-4.875776	-0.816589	0.001424
6	-4.414766	-2.130919	0.000998
1	1.890164	-3.331101	-0.001633
1	-0.835839	-3.842706	-0.001201
1	-4.168945	1.242302	0.001211
1	-5.933242	-0.579797	0.002039
1	-5.101758	-2.973978	0.001245
46	-0.958338	1.260566	-0.000262
7	1.204098	-1.263684	-0.000775
7	-0.901977	-1.672772	-0.000474
7	-2.601817	-0.093504	0.000320
7	-3.110988	-2.433053	0.000344
6	2.498720	-0.548571	-0.000195
6	3.302988	-0.855503	1.272651
6	3.304297	-0.855283	-1.272264
1	2.223208	0.510263	-0.000269
6	4.625290	-0.070263	1.269636
1	3.518175	-1.933515	1.330430
1	2.703472	-0.597269	2.153543
6	4.626605	-0.070016	-1.267672
1	3.519583	-1.933271	-1.330041
1	2.705719	-0.596868	-2.153743
6	5.446496	-0.350427	0.001367
1	5.205549	-0.321733	2.165899
1	4.400900	1.003377	1.329929
1	5.207780	-0.321303	-2.163397
1	4.402247	1.003621	-1.328005
1	6.360205	0.256646	0.001892
1	5.769256	-1.402706	0.001438
17	0.918429	2.687848	-0.000828
6	-2.173208	2.923387	-0.000697
1	-2.799971	2.886598	-0.902869
1	-2.799288	2.887767	0.901986
1	-1.579491	3.837599	-0.001508

11, Isomer **A**:

6	-4.067062	-0.569141	1.198757
6	-2.831489	0.088514	1.242823
6	-0.925645	2.540547	0.052296
6	0.376499	2.899555	0.059555
6	-4.682056	-0.867392	-0.014084
6	-2.237962	0.427420	0.011149
6	4.251226	0.119587	0.002730
6	2.492570	1.578647	0.033062
6	0.263461	0.593259	0.014322
6	5.126647	1.205008	0.023226
6	4.557557	2.477170	0.048992
6	-4.072137	-0.512719	-1.214099
6	-2.835608	0.144464	-1.232328
1	-4.550672	-0.855647	2.128090
1	-1.825690	3.134388	0.063808
1	0.869686	3.857028	0.078245
1	4.561846	-0.922753	-0.018419
1	6.201120	1.063101	0.019257
1	5.176004	3.371707	0.066231
1	-4.559944	-0.754257	-2.153957
46	1.259416	-1.166007	-0.023735
7	-0.979400	1.140148	0.024501
7	1.094443	1.706586	0.036469
7	2.927961	0.313539	0.007970
7	3.231601	2.680618	0.054128
1	-5.639871	-1.380577	-0.024024
6	-2.183222	0.399080	2.589172
1	-1.229110	0.900395	2.402467
6	-2.193630	0.518765	-2.565286
1	-1.229600	0.993433	-2.360236
6	-1.905518	-0.727056	-3.425574
1	-1.256319	-1.429883	-2.894753
1	-2.828644	-1.252920	-3.695524
1	-1.405393	-0.434356	-4.356173
6	-3.054826	1.540701	-3.334462
1	-3.231497	2.445864	-2.741808
1	-2.554481	1.835723	-4.264094
1	-4.032240	1.121942	-3.601310
6	-3.052704	1.362548	3.421468
1	-4.018496	0.912220	3.678759
1	-2.545233	1.618070	4.358904
1	-3.253876	2.293953	2.879420
6	-1.867499	-0.888061	3.375694
1	-2.780168	-1.443689	3.621001
1	-1.210047	-1.548663	2.802340
1	-1.365629	-0.639842	4.318235
6	-0.304657	-2.470383	-0.060446
1	-0.099646	-3.201156	0.726093
1	-1.274798	-1.997863	0.087573
1	-0.259338	-2.964748	-1.035976
17	2.786059	-3.007911	-0.071507

11, Isomer **B**:

6	4.021151	0.360351	1.171225
6	2.734748	-0.183878	1.262291
6	0.666239	-2.613117	0.283907
6	-0.659105	-2.876932	0.310042
6	4.663136	0.491372	-0.056658
6	2.123683	-0.595410	0.063568
6	-4.314434	0.184405	-0.026962
6	-2.655474	-1.382751	0.143105
6	-0.372279	-0.597935	0.061265
6	-5.265904	-0.825605	0.077228
6	-4.791128	-2.127662	0.216758
6	4.024645	0.088427	-1.225733
6	2.737688	-0.461441	-1.195346
1	4.520868	0.695952	2.075237
1	1.519507	-3.268930	0.357629
1	-1.219299	-3.791545	0.409666
1	-4.581070	1.229666	-0.137992
1	-6.325855	-0.601659	0.050640
1	-5.468856	-2.973499	0.304980
1	4.527013	0.211204	-2.180837
46	-1.369984	1.278818	-0.134636
7	0.820754	-1.226628	0.132554
7	-1.282489	-1.634821	0.172701
7	-2.999803	-0.082039	0.005098
7	-3.483880	-2.413261	0.250270
6	-2.602012	2.921251	-0.294708
1	-3.219988	2.796925	-1.195238
1	-3.235835	2.959234	0.602674
1	-2.016924	3.837513	-0.372157
17	0.482229	2.711156	-0.282018
1	5.660995	0.919308	-0.103684
6	2.048006	-0.283276	2.620189
1	1.056829	-0.722754	2.471774
6	2.053563	-0.861208	-2.498509
1	1.063130	-1.259585	-2.257719
6	1.835963	0.367225	-3.404504
1	1.287264	1.150744	-2.872860
1	2.791133	0.790032	-3.738131
1	1.268004	0.081702	-4.298430
6	2.827832	-1.973606	-3.232151
1	2.947724	-2.863612	-2.602934
1	2.295079	-2.270997	-4.143156
1	3.828896	-1.638824	-3.527913
6	2.818450	-1.207233	3.583408
1	3.819816	-0.817093	3.799942
1	2.283715	-1.294268	4.536670
1	2.937230	-2.214831	3.167469
6	1.832403	1.115369	3.232283
1	2.788105	1.597499	3.470871
1	1.289619	1.764736	2.538455
1	1.259186	1.034984	4.164071

12, Isomer **A**:

6	3.770070	-0.982922	1.050532
6	2.437295	-1.391482	1.095322
6	-0.080681	-2.924391	0.061532
6	0.240032	2.049291	0.388874
6	-1.425279	-3.035675	0.013237
6	4.225902	-0.266530	-0.053988
6	1.581338	-1.080374	0.037727
6	-4.723352	0.404828	-0.183578
6	-3.262997	-1.348656	-0.082508
6	-0.896112	-0.788823	0.033605
6	-5.780581	-0.503783	-0.223864
6	-5.453863	-1.858243	-0.185861
6	3.374886	0.048795	-1.111529
6	2.045226	-0.366848	-1.067693
1	4.440777	-1.214269	1.870280
1	2.061515	-1.935816	1.956406
1	0.870950	1.468421	1.066138
1	0.005553	3.025680	0.814379
1	0.693418	-3.675220	0.070220
1	-2.084686	-3.887034	-0.014359
1	-4.840835	1.486108	-0.197767
1	-6.809850	-0.169026	-0.280094
1	-6.223784	-2.625888	-0.212546
1	0.731433	2.168087	-0.581613
1	3.742890	0.609368	-1.963310
1	1.367485	-0.132612	-1.881471
35	6.064961	0.294860	-0.120563
17	-2.706391	3.208263	0.124001
46	-1.547993	1.120829	0.087951
7	0.227801	-1.554687	0.065781
7	-1.912435	-1.731118	-0.009069
7	-3.458711	-0.025474	-0.116691
7	-4.188574	-2.298381	-0.113048

12, Isomer **B**:

6	3.757334	1.414218	-0.794669
6	2.415504	1.790442	-0.800816
6	-0.208837	2.928380	0.072362
6	-1.555743	3.003912	0.092833
6	4.165272	0.370050	0.032104
6	1.502797	1.127664	0.024172
6	-4.800026	-0.488986	0.008487
6	-3.333784	1.263915	0.069154
6	-0.960587	0.768619	0.042888
6	-5.861532	0.408499	0.061116
6	-5.540816	1.762953	0.114782
6	3.256835	-0.304879	0.842278
6	1.916625	0.079267	0.844415
1	4.470228	1.921837	-1.434467
1	2.081818	2.583073	-1.463730

1	0.539699	3.703592	0.107150
1	-2.237777	3.836673	0.126822
1	-4.945784	-1.562199	-0.038628
1	-6.888307	0.062071	0.058466
1	-6.311174	2.529288	0.155625
1	3.581427	-1.128565	1.467365
1	1.199475	-0.449207	1.459062
35	6.020561	-0.149849	0.046224
46	-1.750894	-1.247801	-0.067664
7	0.137044	1.566799	0.041276
7	-2.000167	1.680849	0.074761
7	-3.524050	-0.071595	0.010353
7	-4.275195	2.197290	0.119534
6	-2.819129	-2.998849	-0.193656
1	-3.427790	-3.083975	0.717069
1	-3.459642	-2.935391	-1.083964
1	-2.153410	-3.856968	-0.275639
17	0.200341	-2.549488	-0.147918

XYZ coordinates of calculated structures (B3LYP/6-311+G(d,p))

9, Isomer **A**:

6	3.324795	-0.664135	-1.290608
6	4.555154	1.076375	-0.000836
6	2.081074	2.413679	-0.000284
6	0.807881	2.849238	0.000029
6	-3.262741	0.412088	-0.000581
6	-4.032558	1.570668	-0.000123
6	3.312150	0.172039	-0.000159
6	-1.380017	1.696977	0.000082
6	-3.352066	2.783168	0.000451
6	0.784871	0.541491	0.000087
6	0.838665	-2.758062	0.001224
6	3.325390	-0.663153	1.290918
1	3.443332	-0.008441	-2.157727
1	2.406053	-1.230578	-1.415440
1	4.166557	-1.360558	-1.272689
1	4.611376	1.705590	-0.892417
1	0.575065	-3.332353	-0.888262
1	2.990266	2.982985	-0.000523
1	0.379776	3.834783	0.000100
1	5.436011	0.431747	-0.001015
1	-3.671991	-0.593663	-0.001006
1	-5.113183	1.526372	-0.000199
1	-3.883487	3.729587	0.000845
1	1.897489	-2.524198	0.001435
1	4.612073	1.706006	0.890406
1	0.574496	-3.331327	0.891222
1	4.167158	-1.359576	1.273189
1	2.406712	-1.229528	1.416542
1	3.444254	-0.006806	2.157499
17	-2.200188	-2.770384	-0.000405
7	-1.928033	0.481980	-0.000509
7	2.061021	1.014667	-0.000164
7	0.022595	1.704570	0.000186
7	-2.015825	2.858859	0.000584
46	-0.435493	-1.148832	0.000067

9, Isomer **B**:

6	3.257530	-0.076317	-1.273434
6	4.178741	1.874323	0.000127
6	1.501612	2.777004	0.000135
6	0.169052	2.973625	0.000053
6	-3.385607	-0.187521	-0.000191
6	-4.351790	0.808743	-0.000326
6	3.113236	0.768564	0.000177
6	-1.753673	1.408789	-0.000113
6	-3.900787	2.123139	-0.000336
6	0.575497	0.702247	0.000014
6	3.257489	-0.076160	1.273899
1	3.137125	0.548654	-2.163287

1	2.529842	-0.886074	-1.295653
1	4.255496	-0.521095	-1.299988
1	4.124667	2.504788	-0.891490
1	2.290990	3.504178	0.000209
1	-0.430353	3.865295	0.000030
1	5.159036	1.394998	0.000131
1	-3.640626	-1.239203	-0.000165
1	-5.405345	0.564892	-0.000417
1	-4.590333	2.961218	-0.000438
1	4.124693	2.504845	0.891706
1	4.255446	-0.520955	1.300532
1	2.529786	-0.885901	1.296200
1	3.137075	0.548924	2.163671
7	-2.076525	0.100306	-0.000079
7	1.736201	1.395877	0.000115
7	-0.388842	1.696984	-0.000014
7	-2.600712	2.427125	-0.000233
46	-0.445198	-1.254152	0.000014
6	-1.713182	-2.880671	0.000147
1	-2.330344	-2.816669	0.903243
1	-2.330230	-2.816931	-0.903044
1	-1.152980	-3.811524	0.000314
17	1.319742	-2.814417	-0.000096

10, Isomer **A**:

6	-1.378587	2.510928	-0.000103
6	-0.084380	2.888786	-0.000141
6	3.822867	0.159949	0.000082
6	2.049624	1.595843	-0.000036
6	-0.168677	0.587865	0.000003
6	4.684055	1.253128	0.000032
6	4.103249	2.516493	-0.000056
1	-2.271995	3.109689	-0.000132
1	0.387213	3.854673	-0.000198
1	4.149478	-0.875834	0.000142
1	5.757684	1.122585	0.000059
1	4.710115	3.416376	-0.000101
46	0.862043	-1.161718	0.000036
7	-1.414047	1.112276	0.000003
7	0.652049	1.708272	-0.000067
7	2.499419	0.337306	0.000049
7	2.777040	2.701786	-0.000093
6	-0.701901	-2.483806	-0.000056
1	-1.305761	-2.344552	0.900361
1	-1.305914	-2.344290	-0.900328
1	-0.259620	-3.477876	-0.000235
17	2.427306	-2.975259	0.000046
6	-2.663379	0.326260	0.000089
6	-3.486590	0.583306	1.271437
6	-3.486386	0.582730	-1.271511
1	-2.336547	-0.710258	0.000362
6	-4.764746	-0.268501	1.267900
1	-3.758301	1.644899	1.329567
1	-2.876773	0.360447	2.151819

6	-4.764558	-0.269050	-1.267801
1	-3.758047	1.644308	-1.330157
1	-2.876424	0.359460	-2.151688
6	-5.598463	-0.035998	-0.000063
1	-5.356180	-0.045083	2.160755
1	-4.490590	-1.328343	1.334038
1	-5.355853	-0.046000	-2.160840
1	-4.490409	-1.328924	-1.333452
1	-6.474519	-0.691591	0.000014
1	-5.979739	0.993674	-0.000314

10, Isomer **B**:

6	1.043951	-2.662162	-0.001488
6	-0.280325	-2.925070	-0.001295
6	-3.906442	0.192716	0.001057
6	-2.271887	-1.405190	0.000076
6	0.014799	-0.643345	-0.000170
6	-4.870449	-0.807010	0.001515
6	-4.416688	-2.120527	0.001128
1	1.879197	-3.340320	-0.002041
1	-0.833770	-3.846513	-0.001630
1	-4.160995	1.245086	0.001239
1	-5.924772	-0.566550	0.002129
1	-5.105417	-2.959225	0.001386
46	-0.955562	1.257811	-0.000192
7	1.201635	-1.270303	-0.000823
7	-0.904472	-1.676032	-0.000479
7	-2.598354	-0.093344	0.000300
7	-3.115544	-2.424675	0.000408
6	2.495788	-0.552792	-0.000184
6	3.299720	-0.855758	1.271343
6	3.300898	-0.855100	-1.271127
1	2.217034	0.503613	-0.000057
6	4.619402	-0.069017	1.268677
1	3.516415	-1.930853	1.331511
1	2.701298	-0.596613	2.149590
6	4.620580	-0.068341	-1.266764
1	3.517682	-1.930156	-1.331671
1	2.703311	-0.595489	-2.149805
6	5.440727	-0.344569	0.001259
1	5.199073	-0.320660	2.162122
1	4.394182	1.001820	1.331576
1	5.201082	-0.319499	-2.159806
1	4.395397	1.002523	-1.329307
1	6.348708	0.266457	0.001833
1	5.769698	-1.392509	0.001129
17	0.922677	2.684560	-0.000373
6	-2.169008	2.932161	-0.001375
1	-2.792646	2.896341	-0.902369
1	-2.791645	2.898491	0.900396
1	-1.572743	3.841752	-0.002768

11, Isomer **A**:

6	-4.065513	-0.539667	1.202295
6	-2.830443	0.114090	1.236996
6	-0.920698	2.544550	0.015267
6	0.378858	2.901556	0.016987
6	-4.677546	-0.856455	-0.004285
6	-2.236135	0.431618	0.003039
6	4.245537	0.114975	0.000937
6	2.494385	1.576510	0.008933
6	0.263321	0.597588	0.003436
6	5.122611	1.195713	0.006529
6	4.560287	2.467547	0.013376
6	-4.066274	-0.524557	-1.207157
6	-2.831215	0.129557	-1.234396
1	-4.551156	-0.810472	2.132469
1	-1.815699	3.141786	0.018859
1	0.866713	3.859384	0.022502
1	4.557080	-0.925503	-0.004621
1	6.194317	1.050069	0.005603
1	5.180183	3.358460	0.018035
1	-4.552506	-0.783416	-2.140440
46	1.253769	-1.161757	-0.006777
7	-0.976829	1.144854	0.007019
7	1.096842	1.708444	0.009780
7	2.925053	0.311964	0.002180
7	3.236620	2.672244	0.014655
1	-5.633609	-1.368060	-0.007185
6	-2.181991	0.441599	2.577873
1	-1.228566	0.935404	2.382448
6	-2.184335	0.475106	-2.571421
1	-1.229559	0.964221	-2.370765
6	-1.875802	-0.788896	-3.395588
1	-1.229336	-1.472280	-2.842274
1	-2.790459	-1.326137	-3.662251
1	-1.367459	-0.516486	-4.325013
6	-3.046736	1.466951	-3.375248
1	-3.248272	2.378237	-2.805327
1	-2.534261	1.751356	-4.298693
1	-4.009520	1.028044	-3.652142
6	-3.044470	1.420230	3.397567
1	-4.005968	0.975859	3.670168
1	-2.530568	1.692178	4.323974
1	-3.248630	2.339323	2.841299
6	-1.870088	-0.833763	3.383097
1	-2.783797	-1.375285	3.644300
1	-1.224458	-1.508883	2.818736
1	-1.359732	-0.574184	4.315089
6	-0.318405	-2.467159	-0.017303
1	-0.174785	-3.109161	0.853295
1	-1.290948	-1.983923	0.010823
1	-0.203263	-3.061173	-0.925721
17	2.769236	-3.016685	-0.018381

11, Isomer **B**:

6	4.000565	0.350646	1.182207
6	2.723445	-0.210552	1.261475
6	0.657973	-2.630238	0.238042
6	-0.665741	-2.888860	0.258122
6	4.640786	0.510952	-0.040142
6	2.119772	-0.605893	0.056526
6	-4.301885	0.199040	-0.023486
6	-2.657485	-1.380913	0.116863
6	-0.370699	-0.610824	0.050283
6	-5.258817	-0.803716	0.062228
6	-4.796504	-2.109208	0.176954
6	4.011145	0.120685	-1.215110
6	2.733277	-0.444327	-1.196501
1	4.494697	0.678513	2.089240
1	1.504420	-3.292049	0.301167
1	-1.223327	-3.804021	0.341279
1	-4.563330	1.245732	-0.115030
1	-6.314765	-0.571427	0.040249
1	-5.479376	-2.949487	0.249273
1	4.513351	0.268154	-2.164116
46	-1.356480	1.277482	-0.114907
7	0.817933	-1.242851	0.111471
7	-1.286190	-1.642774	0.142037
7	-2.991583	-0.077255	0.003089
7	-3.492875	-2.402805	0.204526
6	-2.580353	2.937754	-0.248541
1	-3.196390	2.832262	-1.149306
1	-3.210524	2.965171	0.648301
1	-1.989352	3.848400	-0.311180
17	0.500985	2.703071	-0.243195
1	5.630235	0.953124	-0.078128
6	2.035337	-0.339755	2.615249
1	1.057026	-0.797731	2.456958
6	2.055147	-0.826273	-2.507088
1	1.075907	-1.248586	-2.273062
6	1.810129	0.416304	-3.384602
1	1.250306	1.177034	-2.837319
1	2.753743	0.863078	-3.711426
1	1.244963	0.139819	-4.280081
6	2.846199	-1.906454	-3.268250
1	2.991479	-2.803061	-2.658731
1	2.311722	-2.197439	-4.177424
1	3.833976	-1.544193	-3.566793
6	2.818361	-1.258947	3.571248
1	3.805771	-0.849310	3.802312
1	2.277885	-1.371371	4.515800
1	2.963701	-2.255051	3.143303
6	1.786548	1.044466	3.244770
1	2.728596	1.540775	3.496342
1	1.236628	1.693002	2.560200
1	1.209822	0.938755	4.168885

12, Isomer **A**:

6	3.750850	-0.954149	1.066933
6	2.420964	-1.364543	1.108630
6	-0.091527	-2.931679	0.052570
6	0.263738	2.038225	0.374179
6	-1.433818	-3.037970	0.008967
6	4.215049	-0.270922	-0.053516
6	1.577344	-1.090471	0.035258
6	-4.712145	0.418994	-0.171789
6	-3.265858	-1.340919	-0.075378
6	-0.897233	-0.795998	0.039331
6	-5.773308	-0.480582	-0.215622
6	-5.457983	-1.834468	-0.181862
6	3.375583	0.007190	-1.128817
6	2.049346	-0.411115	-1.083790
1	4.411407	-1.155739	1.899773
1	2.038713	-1.880983	1.981240
1	0.911455	1.445113	1.019473
1	0.036233	3.000785	0.827872
1	0.675857	-3.685925	0.057049
1	-2.090287	-3.888635	-0.021646
1	-4.826648	1.498918	-0.184986
1	-6.797894	-0.138780	-0.271749
1	-6.231418	-2.595216	-0.211566
1	0.726632	2.183829	-0.603947
1	3.748846	0.543046	-1.991353
1	1.379874	-0.200697	-1.908113
35	6.041166	0.295059	-0.116551
17	-2.671231	3.220027	0.108856
46	-1.537465	1.114343	0.088451
7	0.222192	-1.563744	0.064346
7	-1.917962	-1.732166	-0.004339
7	-3.452421	-0.018547	-0.104808
7	-4.196852	-2.280199	-0.109856

12, Isomer **B**:

6	3.734835	1.383759	-0.828842
6	2.396431	1.764187	-0.834002
6	-0.227972	2.941616	0.066510
6	-1.572798	3.008440	0.085623
6	4.147280	0.374433	0.036365
6	1.493623	1.144013	0.028638
6	-4.781126	-0.518051	0.015699
6	-3.339204	1.250624	0.068476
6	-0.964061	0.781274	0.043838
6	-5.851383	0.364785	0.065536
6	-5.549767	1.720573	0.112973
6	3.247291	-0.259932	0.885553
6	1.911498	0.130303	0.884017
1	4.440171	1.857468	-1.498557
1	2.058559	2.527977	-1.524532

1	0.511630	3.722361	0.094408
1	-2.254343	3.838782	0.117194
1	-4.916093	-1.591226	-0.026951
1	-6.871513	0.005946	0.065245
1	-6.328263	2.475560	0.150674
1	3.574332	-1.058100	1.538088
1	1.199014	-0.368859	1.525005
35	5.988334	-0.153245	0.048149
46	-1.730463	-1.243370	-0.066586
7	0.126695	1.582791	0.039938
7	-2.010478	1.682699	0.072815
7	-3.512728	-0.085519	0.014713
7	-4.290896	2.168273	0.115176
6	-2.775878	-3.017488	-0.193623
1	-3.374707	-3.113484	0.719235
1	-3.418895	-2.960320	-1.079107
1	-2.097507	-3.861924	-0.281481
17	0.240378	-2.515559	-0.161209
